# Targeting molecular and genetic pathways driving tumorigenesis for precision therapy in colorectal cancer

**DOI:** 10.1080/15384047.2026.2683096

**Published:** 2026-07-26

**Authors:** Allison S. Doermann, Adi Caspi, Andrew E. Evans, Andrea Feci, Ariana A. Entezari, Adam E. Snook, Scott A. Waldman

**Affiliations:** a Department of Pharmacology, Physiology, and Cancer Biology, Thomas Jefferson University, Philadelphia, PA, USA; b Department of Surgery, Thomas Jefferson University Hospital, Philadelphia, PA, USA

**Keywords:** Colon cancer, colorectal cancer, p53, Wnt/β-catenin, GUCY2C, MAPK/ERK, P13K/AKT, SMAD4/TGF-β

## Abstract

Colorectal cancer (CRC) remains a major global health challenge and a leading cause of cancer-related mortality worldwide. Central to its pathogenesis is the dysregulation of interconnected signal transduction networks that govern cellular proliferation, survival, differentiation, apoptosis, and immune modulation. Progressive disruption of these pathways facilitates malignant transformation of the colonic epithelium and underlies disease initiation and progression. In this review, we examine the foundational signaling mechanisms implicated in CRC, with an emphasis on the Wnt/β-catenin, GUCY2C, MAPK/ERK, p53, PI3K/AKT, and SMAD4/TGF-*β* pathways. We also highlight the genomic and epigenomic hallmarks of CRC to illustrate how genetic and epigenetic alterations contribute to diverse oncogenic processes. Furthermore, we discuss both conventional and emerging precision-based therapeutic strategies aimed at driving mutations in CRC tumorigenesis. By synthesizing advances in signaling biology, this review aims to provide a framework for understanding CRC complexity and to inform the development of more precise and effective therapies.

## Introduction

Colorectal cancer (CRC) poses a substantial threat to public health and consistently ranks as the third most prevalent cancer, with 158,850 cases projected in the United States and as the second most deadly, with 55,230 projected deaths in 2026.^1^ One study estimated an additional burden of 5,000 or more excess deaths from CRC by 2040 due to decreased screening during the COVID pandemic.^2^ Further, the incidence of CRC among individuals younger than 50 y has risen steadily since the mid-1990s in the US and other high-income countries, making it the leading cause of cancer-related death in that demographic.^3^ Together, these data underscore the urgent need to understand the molecular mechanisms underlying CRC development and progression to improve prevention and treatment strategies.

Multiple factors play a role in CRC pathogenesis. For example, well-known lifestyle factors include a sedentary lifestyle; a diet rich in calories, fat, and red meat; and smoking.^3^ However, disruption of gene structure and function resulting in dysregulation of physiologic signaling pathways produces tumorigenesis by impacting cell proliferation, apoptosis, and differentiation as well as genomic instability. Many pathways play a role, with the most significant being the Wnt/β-catenin, GUCY2C, MAPK/ERK, p53, PI3K/AKT, and SMAD4/TGF-*β* pathways.^4^ Alterations in gene expression caused by epigenetic changes and/or inherited familial gene mutations also contribute to the formation of colorectal tumors, often by causing aberrancies in those pathways. The complex signaling networks generated by these changes shape the malignant phenotype, modulate the immune response, and structure the tumor microenvironment (TME), ultimately impacting therapeutic efficacy.

With advances in research technologies and genetic profiling in recent years, our understanding of the molecular pathways involved in CRC has broadened. Likewise, the development of precision medicine has led to a paradigm shift in investigating treatments targeting specific gene mutations and molecular alterations for various pathologies, and investigators are exploring these individualized treatments for CRC. As intricate oncogenic signaling networks are revealed and the therapeutic landscape evolves, the goal is to move from the broad approach of traditional chemotherapy to targeted precision oncology. This emerging shift holds the potential to refine CRC management and improve patient outcomes, marking a new era in cancer care on a global scale. Here, we summarize key signaling pathways dysregulated in CRC and their roles in tumor initiation, progression, and therapeutic response. Further, we highlight current and emerging mechanism-based treatment strategies to improve patient outcomes (see Table 1).

**Table 1.** Summary of clinical therapeutic targets for the treatment of colorectal cancer (CRC).

Signaling pathway	Therapeutic target	Agent	Stage of development	References
GUCY2C	GUCY2C	Oral peptide GUCY2C (eg Linaclotide)	Minimal impact (does not make it to colon)	[5]
	GUCY2C	CAR-T cells/CAR-NK cells	Decrease in CRC growth in murine models with metastatic colon cancer; currently in Phase I Clinical trials	[6,7]
	GUCY2C	Vaccine	Phase IIa trial (NCT04111172)	[8]
Wnt/β-catenin	*β*-catenin/CBP	PRI-724	entered phase II clinical trials	[9]
	Tankyrase	RK-287107	Antitumor effects seen in mice	[10,11]
	Porcupine	CGX1321	Phase I Trials	[12]
	*β*-catenin/TCF4 transcriptional complex	TNIK	Preclinical	[13]
	*β*-catenin	ST316	Phase I/II trials (well-tolerated safety profile in patients with advanced tumors) NCT05848739	
p53	Mutant p53	PRIMA-1MET	Phase III clinical trials	[14]
	P53 and AKT	COTI-2	Phase I clinical trials	[15]
	Mutant p53	RETRA	Preclinical	[16]
	MDM2	SA53-OS	Numerous Phase I trials	[17]
	MDM2	RAIN-32	Completed Phase II Trial, short-lived tumor reduction	[18]
	MANIO	p53	Preclinical	[16]
MAPK/ERK	cetuximab/panitumumab	EGFR	Clinical trials have demonstrated effective treatment in some patients	[19–21]
	Vemurafenib/Dabrafenib/Encorafenib	BRAF	Phase II trials- low response rates as monotherapy, mild improvement with addition of MEK inhibitors; good response with triple regimen including EGFR and MEK inhibitors	[22,23]
	PF-07284892	SHP2	Good response clinically combined with encorafenib + cetuximab	[24]
	sotorasib/adagrasib	KRAS	Modest response with monotherapy, improved responses when combined with EGFR inhibitor	[25]
	ulixertinib	ERK 1/2	Acceptable toxicity profile in phase I trials	[26]
	binimetinib	MEK	MEK inhibition lifts the negative feedback on EGFR and triggers its hyperactivation, restoring downstream signaling	[27]
PI3K/AKT/mTOR	alpelisib	PI3K	FDA approved in breast cancer; modest activity for CRC in phase II trial	[28,29]
	capivasertib	AKT	FDA approved in breast cancer; clinical trials ongoing	[30]
	everolimus	mTOR	Modest effect treating CRC in combination with bevacizumab and mFOLFOX-6 in preliminary trials	[31,32]
SMAD/TGF *β*	Vactosertib	TGF-βRI	Phase I/II Clinical Trial; has shown anti-tumor response and a 15.8-month median overall survival, with manageable toxicity	[33,34]
	galunisertib	TGF-βRI	Demonstrated a 32% complete response rate and well-tolerability when added to neoadjuvant regiment in locally advanced rectal cancer in a single-arm Phase II trial	[33,34]
	Bintrafusp alfa	TGF-*β*-binding, TGF-βRII-based fragment and anti-PD-L1	Minimal efficacy in Phase I Trial	[35]
	NIS793	TGF-β	Halted Phase II Trial due to safety concerns NCT04952753	
	GFH018	TGF-βRI	Safe toxicity profile in a Phase I trial in patients with solid tumors	[36]
FAP/MMR	NSAIDS	COX-2	Multiple clinical trials with some treatment effect seen	[37–39]
Epigenetics	DNA methyltransferase inhibitors (DNMTIs)	DNMTs, cytidine analogs	Preclinical success, undergoing clinical trials currently	[40]
	resminostat, belinostat, and panobinostat	HDAC	Undergoing trials (safety profile good but limited efficacy in one trial)	[41]
	APN401	Cbl-b	recently completed trial in patients with advanced disease	[42]
	siRNA	PD1	Currently in clinical trial	[42]
	NBF-006	GSTP	exhibited favorable anti-tumor efficacy in a pre-clinical lung model and recently completed a phase I clinical trial in lung, pancreatic, and CRC patients	[43]

## Microsatellite instability/MMR/FAP

### Background

The average lifetime risk of developing CRC in the United States is approximately 1 in 25.^44^ However, individuals with conditions such as Lynch syndrome (LS; also called hereditary nonpolyposis CRC (HNPCC)) and familial adenomatous polyposis (FAP)) have a significantly greater risk of developing CRC during their lifetime.

### Lynch syndrome

LS is the most common form of hereditary CRC, accounting for approximately 5%–10% of all cases.^45^ For these patients, adenomas commonly develop by the age of 42. In addition to an increased risk of developing CRC, individuals with LS also have an increased risk of developing endometrial cancer.^46^ Disease reflects an inherited mutation in one of the DNA mismatch repair (dMMR) genes, MLH1, MSH2, MSH6, or PMS2, or a mutation in EPCAM, which silences MSH2.^47-51^ Owing to mutations in the MMR genes, LS patient CRC adenocarcinomas are classified as microsatellite instability-high (MSI-H). Microsatellites are tandem repeats of DNA. In MSI-H tumors, mutations in the MMR DNA repair enzymes result in novel accumulation of microsatellites, which can be detected by PCR.^52^ MSI tumors account for approximately 15% of CRCs; in non-LS patients, MSI-H tumors are typically a result of hypermethylation of the MLH1 promoter.^53^

MSI-H tumors have increased lymphocyte infiltration, making the tumor immunologically “hot” compared to MS stable (MSS) tumors.^54^ Additionally, MSI-H tumors encompass lymphocytes that have increased immune checkpoint proteins such as PDL-1, CTLA4, and LAG-3.^55^ Owing to these differences in the tumor microenvironment between MSI-H and MSS tumors, MSI-H tumors tend to respond more favorably to immune checkpoint inhibitors. NCCN guidelines suggest the use of first-line immune checkpoint inhibitors for stage IV CRC (NCCN).^56^ Furthermore, MSI-H tumors have a better stage-adjusted prognosis compared to MSS tumors, despite not responding well to the typical first-line therapy option, 5-FU.^54^ ^,^ ^57^

### Familial adenomatous polyposis

Individuals with FAP inherit a mutated copy of the tumor suppressor Adenomatous Polyposis Coli (APC). Through a loss of heterozygosity, colonic cells lose the regulation of the Wnt/β-catenin signaling pathway. Patients with FAP carry an approximately 100% lifetime risk of developing CRC. FAP patients develop hundreds to thousands of adenomas within their colon. Typically, FAP patients begin receiving flexible sigmoidoscopy or colonoscopy starting at age 10–12.^58^ Often, FAP patients receive a total colectomy in their 20s.^59^ One variant of FAP is Attenuated Familial Adenomatous Polyposis (AFAP), which reflects a mutation in exon 9 of the APC gene. Phenotypically, AFAP is less aggressive than FAP; individuals with AFAP typically develop 10–100 adenomas in their 40s. The lifetime risk of CRC for AFAP is 70%. Unlike in FAP patients, a full colectomy is not recommended for all AFAP patients.^60^ When CRC develops in FAP and AFAP patients, systemic therapy follows the standard-of-care protocols applied to sporadic CRC. Surgical management may vary owing to the underlying polyp burden, while adjuvant chemotherapy decisions remain stage-based and align with those for sporadic cases (NCCN).

### Therapeutic strategies

Owing to the heightened risk of CRC development in patients with FAP and LS, CRC chemoprevention is an active area of research. One approach to chemoprevention is the inhibition of cyclooxygenase-2 (COX-2) by Nonsteroidal Anti-Inflammatory Drugs (NSAIDs). COX-2 is often overexpressed early in CRC tumorigenesis and is the rate-limiting enzyme in prostaglandin production. COX-2 inhibition prevents prostaglandin E2 production, thereby limiting inflammation and, consequently, CRC tumorigenesis.^61^

In LS, the CAPP-2 trial demonstrated that 600 mg of aspirin reduces CRC incidence.^37^ For FAP patients, both celecoxib and sulindac reduce the tumor burden.^39^ ^,^ ^62^ More recent efforts have combined sulindac with various agents to further reduce FAP patient tumor burden. A recent clinical trial combined sulindac with the EGFR inhibitor, erlotinib; however, patients suffered from various adverse events, including nausea, rash, and upper abdominal pain.^39^ Another clinical trial combined sulindac with eflornithine; however, the combination did not further reduce the tumor burden.^63^

While COX-2 inhibition has demonstrated efficacy in reducing adenoma burden and delaying colorectal tumor progression in both FAP and LS patients, there are no FDA-approved chemoprevention therapies for either of these patient populations.^38^ The lack of FDA approval can be attributed to numerous challenges in chemoprevention studies, including surrogate endpoints such as adenoma formation rather than colorectal cancer incidence or mortality, the study durations required for definitive chemoprevention analysis, and stringent safety standards when evaluating agents in otherwise healthy individuals. Notably, COX-2 inhibitors are associated with cardiovascular and gastrointestinal toxicities that limit their suitability as broad chemoprevention compounds. Together, these factors have made the development and evaluation of CRC chemoprevention compounds challenging. Future chemoprevention studies must aim to develop therapeutic compounds that significantly reduce the tumor burden, while demonstrating minimal adverse events.

Numerous innovative therapeutic strategies aimed at preventing CRC development are currently being evaluated in preclinical studies and clinical trials. For FAP, compounds are being studied to target various molecular pathways involved in CRC initiation and progression.^64^ In LS, efforts are focused on exploiting neoantigen formation to develop cancer vaccines or immunotherapeutic approaches that enhance immune recognition and clearance of premalignant cells.^65^

### Wnt/APC/β-catenin pathway

### Background

The Wnt pathway is an integral part of many biological and embryological events. The Wnt family consists of multiple cysteine-rich glycoproteins that function in crucial regulatory roles in cell proliferation, migration, cell fate specification, angiogenesis, and cell division.^66^ These pathways can be further divided into canonical β-catenin-related and non-canonical *β*-catenin-independent signaling. Canonical Wnt signaling maintains crypt stem cell compartments and regulates angiogenesis; therefore, aberrancies in this process are linked to many pathologies. Mutations in this pathway are the leading drivers of CRC.

**Figure 1.**
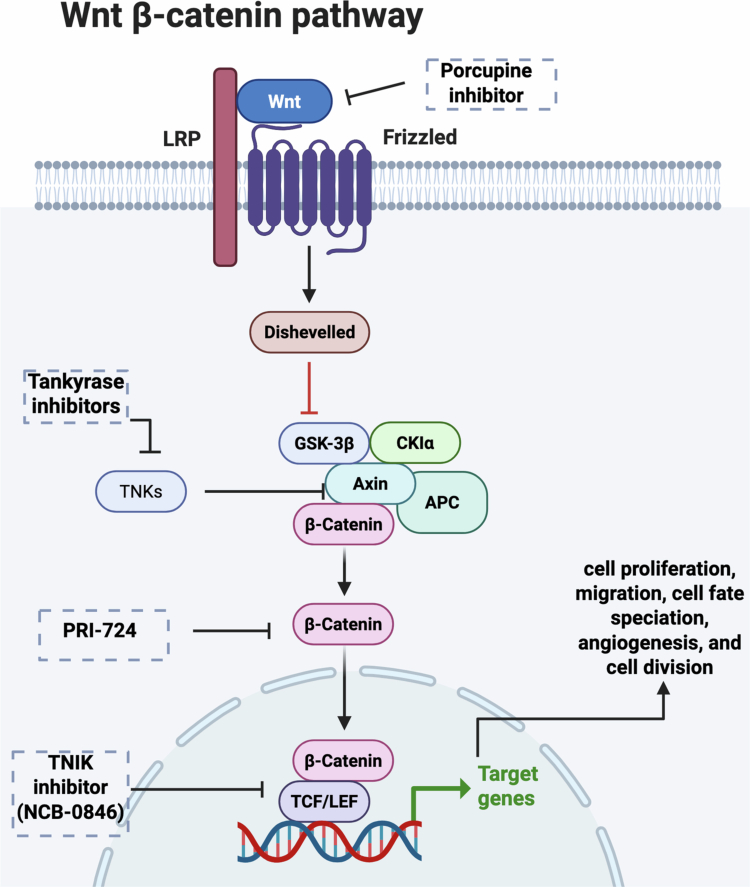
Wnt signaling pathway. Wnt binding to FZD and LRP receptors leads to Dishevelled, disrupting the degradation complex, allowing *β*-catenin accumulation and translocation to activate gene transcription and subsequently cell proliferation, angiogenesis, and cell division. CRC therapeutics are highlighted in dashed boxes with the portion of the pathway they target. Created in BioRender. Doermann, A. (2026) https://BioRender.com/6plv4vc.

The canonical Wnt/β-catenin signaling pathway is initiated when Wnt ligands bind to a core receptor complex composed of LRP5 or LRP6 and members of the Frizzled (FZD) protein family (Figure 1).^67^ In the absence of Wnt ligands, a destruction complex including Axin, CK1α, GSK3β, and adenomatous polyposis coli (APC) phosphorylates *β*-catenin in the cytoplasm. Within this complex, Axin serves as a scaffold that facilitates interactions between GSK3β and APC. GSK3β phosphorylates *β*-catenin, while APC promotes its recognition by the ubiquitin-proteasome system, leading to its degradation. When Wnt ligands are present, they bind to the receptor complex and recruit the cytosolic Dishevelled (Dvl) protein. This interaction disrupts the Axin/GSK3β/APC destruction complex, preventing *β*-catenin phosphorylation and subsequent degradation. Disruption of the destruction complex releases *β*-catenin and allows it to accumulate in the nucleus along with members of the LEF/TCF transcription factor family.^68^ As nuclear concentrations increase, LEF/TCFs recruit *β*-catenin to target genes and nucleate its associations with co-regulators Pygopus, BCL9/Legless, and transcription-activating complexes such as the CDK8 module of Mediator and TRRAP. Activated Wnt signaling and the subsequent translocation of *β*-catenin lead to the transcription of CRC-associated genes such as MYC.^69^

### Mutational landscape

Due to the Wnt pathway’s involvement in early gut development, it has been investigated for its involvement in human cancer, and mutations in this pathway can consequently lead to CRC development. The Wnt/β-catenin pathway is mutated in up to 93% of CRCs, with APC mutations being the most common. APC mutations typically result in an APC protein truncation and loss of *β*-catenin binding function, which results in *β*-catenin stabilization. An additional 5% of tumors exhibit activating mutations in *β*-catenin.^4^ ^,^ ^34^ These mutations allow the Wnt cascade to continue signaling aberrantly, even in the absence of Wnt ligands.

### Therapeutic strategies

Because CRC tumors have a high frequency of Wnt/β-catenin mutations, this pathway is a compelling therapeutic target. Direct small-molecule inhibitors of *β*-catenin remain challenging due to the protein’s structure; however, in recent years, several compounds have entered the clinical phase. ST316 is a *β*-catenin antagonist currently in a Phase I/II clinical trial (NCT05848739) following a well-tolerated safety profile in patients with advanced tumors. Additionally, progress has been made in the development of compounds targeting *β*-catenin/TCF interactions. For example, PRI-724, a *β*-catenin/CBP antagonist, has entered clinical trials (NCT01302405 and NCT02413853), where it has demonstrated acceptable toxicity profiles.^9^ However, more recent work focuses on upstream pathway inhibition rather than direct *β*-catenin targeting. Tankyrase (TNK) inhibitors (RK-287107) stabilize Axin and promote *β*-catenin degradation.^10^ ^,^ ^11^ Porcupine inhibitors such as CGX1321 function in CRC by blocking Wnt ligand secretion and may have therapeutic benefit.^12^ TNIK inhibitors, which disrupt the *β*-catenin/TCF4 transcriptional complex, are in preclinical development, with evidence supporting their ability to block Wnt signaling in APC-mutant cells.^13^ Finally, recent studies focus on restoring wild-type APC expression in CRC.^70^ Logistical challenges are also posed by targeting Wnt signaling. Wnt pathway activation occurs very early in disease progression, often considered to be a hallmark of transformation; in more advanced tumors, the mutational burden and signaling complexities have often progressed beyond Wnt to include MAPK, p53, etc., and tumors become less exclusively reliant on Wnt signaling, impacting their susceptibility to Wnt-targeted therapies.^68^ Therefore, targeting Wnt/β-catenin may be more effective in early-stage disease or as part of a combinatorial approach.

## Guanylyl cyclase C (GUCY2C) signaling pathway

### Background

Guanylyl cyclase C (GUCY2C) is an intestine-specific transmembrane receptor activated by the endogenous guanylin and uroguanylin, or exogenous bacterial heat-stable enterotoxins, which functions as a tumor suppressor by restricting proliferation, maintaining genomic integrity, and promoting differentiation.^71-73^ Ligand-activated GUCY2C catalyzes the synthesis of intracellular cyclic GMP (cGMP) from GTP, initiating signaling cascades underlying homeostasis of the intestinal epithelium. This signaling axis participates in various functions regulating fluid secretion, microbiome composition, intestinal barrier integrity, epithelial renewal, cell cycle progression, responses to DNA damage, epithelial‒mesenchymal crosstalk, cell migration, and the cellular metabolic status. Elimination of GUCY2C signaling in mice increased susceptibility to intestinal tumorigenesis induced by carcinogens or inherited germline mutations, while supplementation with uroguanylin decreases tumorigenesis.^72^ Dysregulation of the GUCY2C-cGMP signaling axis has been implicated in the pathogenesis of bowel transit disorders, inflammatory bowel disease, and CRC.

#### Mutational landscape

GUCY2C mutations are present in 5% of CRC, but no specific CRC-related mutations have been identified; guanylin and uroguanylin mutations occur in fewer than 2% of tumors.^74^ Instead, in CRC tumorigenesis, GUCY2C ligands guanylin and uroguanylin are two of the more commonly suppressed genes; specifically, their gene expression is suppressed by activation of the Wnt signaling pathway, which silences GUCY2C signaling.^73^ ^,^ ^75^ ^,^ ^76^ This highlights a critical connection between GUCY2C signaling and early tumorigenic development induced by Wnt/β-catenin and underscores GUCY2C’s role as a tumor suppressor. Deficiency in GUCY2C signaling accelerates tumorigenesis in the context of APC mutations in mice by enhancing proliferation and reducing genomic stability. While guanylin and uroguanylin expression is reduced in tumors, GUCY2C expression is retained in primary tumors, metastatic tumors, and non-intestinal tumors arising from intestinal metaplasia.^77^ ^,^ ^78^ This finding positions GUCY2C as a potential therapeutic target, both in the context of chemoprevention and immunological targeting.

#### Therapeutic strategies

GUCY2C’s function as a tumor suppressor, coupled with its retained expression in tumors, poses a potential avenue for CRC chemoprevention. Reactivation of the signaling pathway via ligand replacement may allow for continued tumor-suppressive effects, even in the absence of endogenous guanylin and uroguanylin. In preclinical models, the administration of GUCY2C agonists opposed tumorigenesis.^79^ ^,^ ^80^ The FDA-approved GUCY2C agonist linaclotide was evaluated in a clinical setting; however, it demonstrated limited bioavailability in the colon and was primarily absorbed in the small intestine.^5^ An active clinical trial is also evaluating its efficacy in CRC patients to better characterize its impacts on CRC-related markers, including guanylin and GUCY2C activity (NCT03796884).

As GUCY2C expression is retained in advanced and metastatic tumors, and its expression in healthy intestine is apically restricted, it has also been exploited as a CRC antigen for immunotherapy. A GUCY2C-targeted cancer vaccine was well-tolerated in a Phase I study and is currently being evaluated in a Phase II setting (NCT04111172), as well as a Phase I trial with a Listeria-derived vector (NCT07417488).^8^ This presents an opportunity to treat existing tumors and metastases, as well as prevent disease in patients at risk of recurrence.

Additionally, engineering chimeric antigen receptor (CAR) T cells targeting GUCY2C has been developed to treat advanced CRC and has been effective in the preclinical and clinical setting.^6-8^ A CD3ε-GUCY2C bispecific antibody was also recently evaluated in a clinical trial, though the study was terminated owing to strategic considerations (NCT04171141). Further, GUCY2C is being evaluated as a target for bispecific CAR-T cell therapy (NCT07152210) as well as for CAR-NK (natural killer cell) therapy (NCT07462650). Overall, the GUCY2C pathway serves as a uniquely promising target in CRC across the spectrum of disease progression, from chemoprevention to treatment of advanced tumors.

## p53

### Background

p53 is a tumor suppressor that is frequently mutated in CRC. Cellular stress activates p53, which regulates apoptosis, DNA repair, and cell cycle arrest. p53 primarily acts as a transcription factor controlling the expression of hundreds of target genes.^16^ In healthy cells, p53 levels are maintained primarily through MDM2-mediated ubiquitination, a process enhanced by MDM4; MDM2 and MDM4 are regulated by a negative feedback loop involving p53. In response to stress, such as DNA damage or oncogenic signals, this loop is disrupted through post-translational modifications (PTMs) such as phosphorylation and acetylation, which stabilize and activate p53. Key kinases (ATM, ATR, CHK1/2) phosphorylate p53 and its regulators, promoting p53 activation and the transcription of stress-specific genes. Additional PTMs, such as ubiquitin-like modifiers and isomerization, further regulate p53's function.^81^ Depending on the type and strength of the stress and the cellular context, p53 can trigger outcomes varying from cell survival to apoptosis through its role as a transcription factor. In CRC, p53 regulates the expression of apoptotic genes such as PUMA, BAX, and NOXA. To regulate cell cycle progression, p53 regulates genes like CDKN1A, GADD45A, and SFN.^82^ Specific PTMs, such as Ser46 phosphorylation and Lys120 or Lys320 acetylation, determine whether p53 promotes cell death or survival by modulating target gene selection and protein interactions.^83^

#### Mutational landscape

Mutant p53 is an attractive cancer therapy target, as it is present in over 50% of CRC tumors and is present overall in 50% of all tumor types.^16^ TP53 mutations are commonly missense mutations that impair DNA-binding function, leading to loss of transcriptional tumor suppressor activity and dysregulation of cell cycle progression, apoptosis, and DNA repair.^82^ Interestingly, in addition to LOF mutations, some p53 missense mutations can also lead to Gain-of-Function (GOF) mutations.^84^ These GOF mutations reprogram the transcriptional network within the cell and can increase cell proliferation, invasion, and migration by enhancing signaling through the epidermal growth factor receptor and TGF-*β* receptor.^84^ In addition to missense mutations, TP53 can acquire nonsense or frameshift mutations, resulting in a truncated protein and complete loss of p53 function.^85^ Furthermore, the p53 signaling pathway can also be disrupted by MDM2 overexpression. In CRC, it is estimated that MDM2 is overexpressed in approximately 10% of tumors, which leads to the degradation and loss of p53.^86^

#### Therapeutic strategies

Potential therapeutic strategies include restoring wild-type p53 function using compounds such as PRIMA-1MET and COTI-2, promoting the degradation of mutant p53 via HSP90 or HDAC inhibition, and disrupting gain-of-function (GOF) interactions with proteins such as p63/p73 using small molecules such as RETRA.^14^ ^,^ ^16^ COTI-2 is the only agent used in clinical trials (NCT02433626) for the treatment of CRC, and this is only in a phase I trial.^15^ The other agents have undergone clinical trials for other cancers with some success. Other approaches involve targeting mutant p53-driven pathways (e.g., the mevalonate pathway for cholesterol synthesis with statins), reinitiating the activity of p53, exploiting synthetic lethality by inhibiting kinases such as CHK1 or WEE1, or developing immunotherapies such as vaccines or immune checkpoint combinations.^87^ ^,^ ^88^ For example, mutant p53 regulates the expression of PD-L1 in cancer cells, and PD-1-targeted antibodies, which inhibit the interaction between PD-1 and PD-L1, have demonstrated promising outcomes in treating metastatic, microsatellite instability high (MSI-H) CRC.^89^ ^,^ ^90^ Drug repurposing (e.g., metformin, PARP inhibitors) offers promising fast-track options, reflecting known safety profiles.^91^ However, because mutant p53 variants differ in structure and function, a universal treatment is unlikely to be effective, highlighting the need for individualized mutation-specific strategies.

In addition to targeting p53, research efforts have also focused on targeting MDM2. MDM2 inhibitors are primarily focused on treating tumors with wild-type p53 or rely on impairing MDM2-p53-independent effects to target the tumor. MDM2 targeting has been challenging because the MDM2-p53 interaction is a necessary signaling component in normal cells.^18^ While there are no FDA-approved MDM2 inhibitors in CRC, there is an MDM2 inhibitor with an ongoing Phase I clinical trial for locally advanced or metastatic wild-type p53 tumors, SA53-OS (NCT06578624), and numerous Phase I trials that have been recently completed (NCT02264613, NCT00559533, NCT02935907, NCT01723020, NCT01760525, and NCT02143635).^17^ One MDM2 inhibitor, RAIN-32, recently completed a Phase II trial with a reasonable safety profile; however, tumor reductions were short-lived. While theoretically targeting MDM2 is a promising therapeutic strategy in CRC, more research is needed to develop effective treatment regimens.

## MAPK/ERK pathway

### Background

The mitogen-activated protein kinase (MAPK)/ERK pathway is a serine/threonine kinase signaling cascade that relays extracellular signals from receptor tyrosine kinases (RTKs), most notably EGFR, and propagates the signal through sequential activation of downstream kinases, eventually reaching the genome.^92^ In the normal intestine, MAPK signaling is a key driver of cellular proliferation and differentiation in response to growth factors, hormones, cytokines, and stress.^93^ In CRC, MAPK over-activation promotes cell survival, proliferation, tumor invasion, and metastasis, and is involved in over 60% of CRC cases.^94^ Accounting for the majority of these, mutations in the RAS-family proteins occur in over 50% of CRC patients, with 86% of these being KRAS mutant, while downstream kinase BRAF mutations account for another 10%.^95^ ^,^ ^96^ Both have been extensively characterized as prognostic markers of poorer OS and RFS in advanced CRC. Importantly, the KRAS mutation status serves as a predictive biomarker for a poor response to anti-EGFR therapy. In addition, the long-awaited advent of KRAS inhibitors has transformed this specific marker into a therapeutic target with promising clinical activity in refractory metastatic CRC. Overall, the high prevalence of MAPK/ERK pathway mutations in CRC, together with their established role in determining poor patient outcomes, makes them relevant for therapeutic targeting.

**Figure 2.**
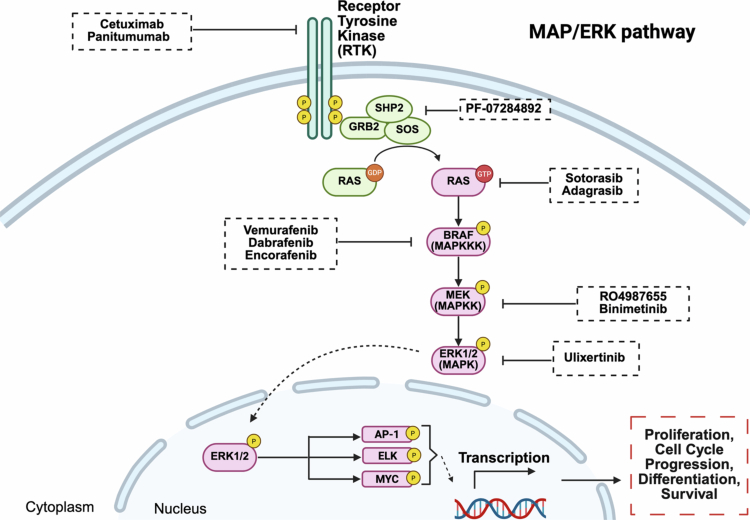
*The MAP/ERK pathway*. The MAP kinase cascade is initiated with the activation of a receptor tyrosine kinase, which leads to the phosphorylation of MAP kinases and ultimately numerous transcription factors in the nucleus. Current CRC therapeutics are highlighted in the boxes with dashed lines, along with the spot in the pathway they target. Created in BioRender. Doermann, A. (2026) https://BioRender.com/6jd2uct.

The MAPK signaling cascade transduces growth factor and other signals from RTK to nuclear effectors. Within the MAPK family, the ERK cascade is the major pathway implicated in CRC, integrating RTK signals through the extracellular-signal-regulated kinases RAS, RAF, MEK, and ERK to regulate nuclear transcription factors. See Figure 2 for a graphical depiction of the pathway. Upon the binding of growth factor or other ligand binding to the RTK, most notably EGFR in CRC, receptor homo- or heterodimerization and autophosphorylation follow. This event leads to the recruitment of adaptor proteins, including GRB2 and the guanine nucleotide exchange factor SOS, which facilitate the GDP to GTP nucleotide exchange on RAS, activating it. Ras includes small GTPases KRAS (KRAS4A and KRAS4B splice variants), NRAS, and HRAS. Active RAS (GTP-bound) then recruits and activates the ser/thr MAPKKK RAF (ARAF, BRAF, and CRAF), which in turn phosphorylates and activates cytoplasm-bound thr/tyr MAPKK MEK1/2. RAS returns to the “off”/GDP-bound state through a slow process of intrinsic hydrolysis, whose rate can be significantly increased by GAPs. In addition, PKC isoforms can enhance RAF activation, and MAP3K8 can bypass RAS to activate MEK directly. Ultimately, MEK1/2 phosphorylates, activates, and separates from ERK1/2, the terminal ser/thr MAPK, which translocates to the nucleus to regulate the transcription factor families AP-1, ELK, and MYC, thereby promoting cell cycle progression, proliferation, differentiation, and survival.^97^ ^,^ ^98^ In CRC, constitutive activation of MAPK/ERK is multifactorial and can occur at different levels: upregulation of EGFR and mutations of KRAS, NRAS, and BRAF. Furthermore, crosstalk with parallel pathways, including the other MAPK subfamilies p38 and JNK, as well as the PI3K/AKT pathway and scaffolding proteins, fine-tunes ERK signaling and can contribute to CRC development and progression.

#### Mutational landscape

In CRC, the predominant mutant KRAS isoform is G12D (28%), followed by G12V (20%), G13D (18%), G12C (7%), A146T (7%), and G12A (4%), with the rest comprising a small minority.^99^ These alterations slow down GAP-mediated and intrinsic GTP hydrolysis, determining a prolonged KRAS “on” status.^100^ Hyperactive KRAS drives persistent MAPK and PI3K pathway signaling, determining uncontrolled proliferation and resistance to apoptosis. These alterations promote tumor growth and support its invasive and metastatic potential.^98^ In colorectal cancer, sustained KRAS signaling allows cells to bypass upstream receptor control, determining the inefficacy of EGFR inhibitors. Consequently, activating KRAS mutations gained a role as a predictive biomarker for primary resistance to the anti-EGFR monoclonal antibodies cetuximab and panitumumab, which are clinically used for metastatic colorectal cancer.^101^ The prognostic implications of carrying KRAS mutations, independent of treatment, clearly point to worse OS and RFS in advanced, as well as earlier-stage, CRC; however, the prognostic interpretation of variant-specific mutations is still unclear and warrants larger studies.^102^ ^,^ ^103^ Immediately downstream of Ras, the effector protein BRAF propagates the MAPK signaling cascade. Binding to GTP-loaded RAS triggers BRAF membrane recruitment and relieves BRAF autoinhibition, leading to BRAF homodimerization or BRAF-CRAF dimerization, which in turn renders it amenable to activating autophosphorylation or phosphorylation by upstream kinases. In its dimeric, phosphorylated state, BRAF becomes fully active and able to phosphorylate MEK.^104^ In BRAF-mutant CRC, 75% of mutants are valine 600 substitutions, mainly to an “on” phosphomimicking glutamate, and far less commonly to lysine.^105^ Clinically, BRAF V600E tumors often present with a characteristic phenotype of mucinous, signet ring, or poorly differentiated histology, right-sidedness, and with perineural and vascular invasion.^106^ Non-V600E mutations, also described as class II and III, result in RAS-independent constitutive activation of BRAF dimers and increased avidity for RAS and CRAF, respectively.^105^ Similarly to KRAS mutants, V600E BRAF mutation also emerged as a predictor of lack of benefit to anti-EGFR therapy and prognosticates inferior PFS and OS.^107^ ^,^ ^108^ Another important consideration is that BRAF mutations are closely associated with mismatch repair-deficient (MMR-D) CRC, occurring in about 35% of these.^109^ Despite its enrichment among MSI-high tumors that generally respond to immune checkpoint inhibitors, the BRAF V600E mutation is associated with non-response and poorer survival outcomes within the MMR-deficient cohort.^110^ Taken together, these findings define BRAF-mutant CRC as a distinct molecular subset and highlight the need for further studies to clarify how different BRAF mutation classes can be effectively targeted.

#### Therapeutic strategies

The therapeutic targeting of MAPK/ERK in CRC began in the early 2000s with monoclonal antibodies targeting EGFR (EGFRi). The CRYSTAL (cetuximab + FOLFIRI) (20), OPUS (cetuximab + FOLFOX4), and PRIME (panitumumab + FOLFOX4) trials established that the addition of an EGFRi to the chemotherapy backbone significantly reduced cancer progression and improved the response rate.^19^ ^,^ ^21^ These studies introduced a paradigm that response is only observed in KRAS-WT patients, and that among KRAS-WT, BRAF-V600E patients, no significant benefit from cetuximab was observed.^20^ Following the successes of BRAF inhibitors (BRAFi) in melanoma, vemurafenib and dabrafenib were evaluated in BRAF V600E-mutant metastatic CRC in a phase II setting. As a monotherapy, response rates are low (5%–11%), survival outcomes are short, and the failure was attributed mechanistically to increased feedback EGFR activity. By the same mechanism, MEK inhibitors (MEKi) showed no meaningful single-agent activity in KRAS-mutant CRC. Slightly better response rates and PFS were achieved by BRAFi plus MEKi combination therapy. Phase II studies of BRAFi plus EGFRi reported similar low-response rates, but stable disease in over 50% of patients. In the triple combination regimen (EGFRi plus BRAFi plus MEKi), the response rate significantly improved compared to the double regimen lacking MEKi.^22^ ^,^ ^23^ In the first phase III trial (SWOG), patients received vemurafenib in addition to irinotecan and cetuximab standard therapy; however, survival was not improved, and the rate of adverse events was higher. The BEACON phase III trial demonstrated that combining BRAFi and EGFRi with or without MEKi in pretreated patients resulted in improved OS and response rate, and more remarkably so in the triple regimen, compared to EGFRi plus cytotoxic agent standard therapy. In the double regimen (BRAFi plus EGFRi) group, high tumor mutational burden (TMB) was identified as the main driver of resistance. This prompted a pilot study of the anti-PD-1 inhibitor nivolumab in addition to the BRAFi plus EGFRi regimen in microsatellite-stable mCRC, which achieved high response and disease control rates. Building on the results from the BEACON trial, the BREAKWATER trial showed that the addition of BRAFi and EGFRi to first-line standard therapy, significantly improved survival outcomes compared to standard therapy, in BRAF V600E-mutated metastatic CRC.^111^ ^,^ ^112^

Following the success of MAPK pathway blockade in BRAF-mutant CRC, KRAS inhibitors are now being clinically evaluated. The covalent KRAS G12C inhibitors sotorasib and adagrasib, despite modest monotherapy activity in CRC, have shown improved responses in combination with EGFRi in the KRYSTAL-1 trial.^25^ This resulted in accelerated FDA approval in June 2024 of adagrasib + cetuximab for previously treated KRAS G12C CRC. Although KRAS G12C inhibitors have demonstrated promising activity, this mutation only accounts for a minority of KRAS alterations in CRC. Emerging KRAS(ON)/pan-RAS inhibitors, which are capable of targeting all WT and mutant RAS isoforms, have achieved notable preclinical efficacy and are currently being evaluated in early-phase trials across several malignancies, including CRC. Attempts at targeting other signaling nodes within the MAPK/ERK pathway have been attempted. SHP2 inhibition is in early clinical testing for solid tumors. SHP2 is a non-receptor protein tyrosine phosphatase required for Grb2/SOS complex formation, and its inhibition is an attractive strategy for halting RTK-driven oncogenic signaling.^113^ SHP2 inhibition prevents adaptive resistance to MEK inhibitors in multiple preclinical models.^114^ In its very first clinical use, SHP2 inhibitor monotherapy failed to control disease in a patient with mutant BRAF metastatic CRC, but re-addition of encorafenib + cetuximab produced a renewed response with PFS three times longer than the prior regimen, suggesting that SHP2 inhibition had at least a role in resensitizing the cancer to targeted therapy.^24^ At the distal end of the cascade, ERK1/2 kinases represent an attractive target that can, in principle, suppress MAPK signaling irrespective of the upstream KRAS or BRAF genotype. The ERK inhibitor ulixertinib demonstrated proof-of-concept activity with manageable toxicity in a phase I study of MAPK-altered solid tumors, including BRAF-mutant CRC.^26^ Resistance mechanisms remain a major source of transient response or failure to MAPK-directed therapies in CRC. BRAF and/or MEK inhibition promotes negative feedback on EGFR and triggers its hyperactivation, restoring downstream signaling.^27^ Additionally, following KRAS G12C inhibition, tumors can acquire secondary RAS (or other) mutations and gene amplifications that reactivate ERK despite upstream inhibition.^115^ Furthermore, under MAPK-targeted suppression, crosstalk with the PI3K/AKT pathway may also sustain tumor cell survival and blunt response to therapy.^116^ Beyond vertical pathway suppression, horizontal targeting of parallel pathways, most notably the PI3K/AKT pathway, is supported by preclinical data and may yield clinical benefit in CRC.^117^ Overall, biomarker-guided combination strategies that target feedback loops and pathway crosstalk represent a key direction for overcoming resistance to MAPK-targeted therapies in CRC.

## PI3k/AKT/mTOR

### Background

**Figure 3.**
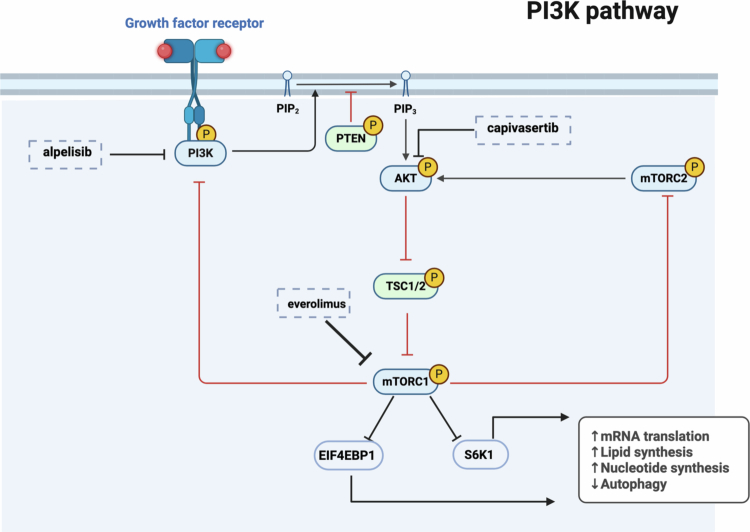
*The PI3K/Akt/mTOR pathway.* The PI3K pathway is triggered by a growth factor binding the receptor and causing the phosphorylation of PI2K to PI3K, leading to Akt activation, followed by mTOR activation and subsequent downstream effector modulation. Created in BioRender. Doermann, A. (2026) https://BioRender.com/7wqc1gf.

The PI3K/AKT pathway is a critical intracellular signaling cascade that regulates cell growth, survival, metabolism, and proliferation [69]. Growth factors bind to receptor tyrosine kinases, activating the class I PI3K, which catalyzes the formation of phosphatidylinositol-3,4,5-trisphosphate (PIP3) from phosphatidylinositol-4,5-bisphosphate (PIP2) and recruits AKT kinase to the plasma membrane.^118^ See Figure 3 for a graphical depiction of the pathway. Once activated, AKT phosphorylates and activates or inhibits multiple downstream targets with pro-tumorigenic or tumor suppressive capacities, respectively.^119^ One downstream target, the mTORC1 complex, is activated through inhibition of its negative regulator tuberous sclerosis complex (TSC) by AKT.^119^ Upon activation, mTORC1 drives cell growth and protein translation by inhibiting translation of the negative regulator EIF4EBP1 and the kinase S6K1.^118^ Further, Phosphatase and TENs in homolog (PTEN) on chromosome 10 negatively regulate PI3K/AKT signaling by dephosphorylating PIP3, silencing the pathway.^118^ ^,^ ^120^ In CRC, the PI3K/AKT pathway is frequently dysregulated through genetic and epigenetic alterations that drive uncontrolled cell growth, survival, and metabolism.^118^

#### Mutational landscape

Mutations in PIK3CA are among the most common in CRC, occurring in ~10–15% of tumors.^121^ Mutations lead to activation of the PI3K catalytic subunit and persistent downstream AKT signaling.^118^ There are recurrent hotspots in the gene in exons 9 and 20, occurring within either the helical or kinase domains.^17^ PTEN loss or inactivation, through mutation, deletion, or promoter methylation, also contributes to oncogenic signaling by removing the pathway’s inhibitory control.^120^ In CRC, PTEN has relatively low mutation rates (~9%), but loss of protein expression is observed in up to one-third of CRC cases.^120^ ^,^ ^122^ ^,^ ^123^ Additionally, aberrant activation of upstream receptors like EGFR or KRAS mutations can further enhance PI3K/AKT signaling, promoting tumor progression and therapeutic resistance.^118^ ^,^ ^124^ ^,^ ^125^ In a meta-analysis, mutations in PIK3CA and non-functional PTEN significantly predicted a poor objective response rate (ORR) (OR = 0.39, and OR = 0.41, respectively).^126^ Finally, AKT mutations are rare in CRC: the most common, E17K, is found in ~0.7% of CRCs.^127^

#### Therapeutic strategies

Given its frequent activation and central role in tumor progression, the PI3K/AKT signaling cascade represents a promising avenue for targeted therapies in CRC.^17^ ^,^ ^118^ ^,^ ^120^ ^,^ ^121^ ^,^ ^123^ Therapeutic interventions designed to inhibit PI3K/AKT signaling aim to suppress aberrant survival and proliferation signals, and a variety of inhibitors have entered clinical trials to assess their efficacy in CRC.

Many preclinical and clinical investigations have assessed the therapeutic potential of targeting the PI3K/AKT pathway in tumor progression. Because the PI3K/AKT/mTOR signaling network contains extensive crosstalk and functional redundancy, combination therapeutic strategies are being explored to circumvent resistance mechanisms and improve treatment responses. Indeed, the combined inhibition of MEK and PI3KCA is an effective antitumor strategy in HER2 gene-amplified CRC.^127^

The PIK3CA-selective inhibitor alpelisib reduces CRC cell migration and invasion in vitro and decreases the metastatic burden in vivo.^28^ Alpelisib is FDA-approved, in combination with fulvestrant, for postmenopausal patients with hormone receptor (HR)-positive, HER2-negative, PIK3CA-mutated advanced or metastatic breast cancer (MBC).^128^ Clinical experience with alpelisib in CRC has been more limited. In a phase II study of patients with PIK3CA-mutant metastatic CRC, alpelisib plus capecitabine demonstrated overall modest activity. However, patients lacking KRAS co-mutations or liver metastases achieved longer progression-free survival and were the only individuals who exhibited objective responses, although frequent hyperglycemia necessitated treatment discontinuation (NCT04753203).^29^

The FDA approved pan-AKT inhibitor capivasertib in combination with fulvestrant for adult patients with HR-positive, HER2-negative, locally advanced, or MBC whose tumors have one or more PIK3CA/AKT1/PTEN alterations (NCT04305496).^30^ Preclinical work suggests that capivasertib may be effective in suppressing tumor growth in CRC, but clinical trials are ongoing (NCT02465060).

Finally, the mTOR inhibitor everolimus has been explored to treat CRC with mixed success.^31^ ^,^ ^129^ ^,^ ^130^ Although everolimus as a single agent did not confer meaningful efficacy in pretreated mCRC patients, everolimus in combination with other agents has had some success.^31^ ^,^ ^129^ ^,^ ^130^ Indeed, bevacizumab plus everolimus has modest activity in patients with refractory mCRC (NCT00597506).^31^ Similarly, everolimus and mFOLFOX-6 + bevacizumab demonstrated preliminary efficacy for first-line mCRC (NCT01047293).^32^

## SMAD/TGF-β

### Background

The transforming growth factor-*β* (TGF-*β*) pathway is a key tumor suppressor signaling cascade in normal intestinal physiology, and its perturbation can affect tumorigenesis. In normal epithelial physiology, extracellular TGF-*β* ligands (TGF-β1/2/3) bind to TGF-*β* receptor II (TGF-βRII). TGF-βRII recruits and phosphorylates TGF-βRI, which in turn phosphorylates receptor-SMAD2/3 and allows them to complex with SMAD4.^131^ The SMAD2/3-4 complex translocases to the nucleus and drives the expression of genes involved in cell cycle arrest, apoptosis and differentiation, particularly via p15 and p21 upregulation, and c-Myc downregulation, acting as a proliferation break in epithelial cells^132^ The TGF-*β* signaling pathway plays a dual role in CRC, initially suppressing tumor formation but later promoting tumor progression upon loss of SMAD4. SMAD4 is a central mediator of this pathway, and its loss disrupts cell cycle regulation and apoptosis, allowing cells to evade growth inhibition and contribute to tumor development.^133^ Also, aberrant SMAD4 function alters the role of the TGF-*β* pathway in shaping the tumor microenvironment, enhancing tumor invasion, migration, and blood vessel formation, and worsening outcomes in CRC patients. While SMAD4 primarily acts in the classical SMAD-dependent pathway, it also has indirect effects on SMAD-independent TGF-*β* pathways involving factors such as TRAF4, TRAF6, and TAK1, which influence apoptosis and cell migration. Beyond transducing the TGF-*β* pathway, SMAD4 also interacts with other signaling pathways, such as the BMP, Wnt/β-catenin, and Notch, influencing processes such as cell proliferation, differentiation, and tissue regeneration. Thus, SMAD4 is crucial not only for proper TGF-*β* signal transduction but also for maintaining the balance between tumor suppression and promotion.

#### Mutational landscape

SMAD4 is one of the most frequently altered genes in CRC, with homozygous loss of expression or function in 10%–38% of CRCs,^134^ and loss of at least one allele in up to 68% (Mamot et al., 2003). Deletion of chromosome 18q21, containing the SMAD4 locus, is the most frequent alteration, followed by missense mutations in the MH2 structural domain, which is critical for SMAD complex formation and nuclear translocation, and, less commonly, MH1 domain (DNA-binding) mutations.^135^. Notably, 18q21 also harbors the locus of co-SMAD SMAD2. These changes do not directly cause tumor aggressiveness but impede the transduction of growth-suppressing TGF-*β* signaling and its transcriptional program. Upon SMAD4 loss, TGF-*β* signaling switches from tumor-suppressive to tumor-promoting through support of epithelial‒mesenchymal transition, immune evasion via TGF-*β*-mediated T and NK suppression, and stromal remodeling.^136^ Overall, SMAD4 loss correlates with disease aggressiveness, worse overall survival and higher rates of metastasis and lymph node involvement.^137^ In addition, SMAD4 loss is a predictive marker for poor response to neoadjuvant 5-fluorouracil-based chemotherapy in CRC. Notably, germline SMAD4 mutations are a major cause of Juvenile Polyposis Syndrome and determine predisposition to CRC.^138^ Beyond SMAD4, other protein members of the pathway are often mutated in CRC. TGFβRII is mutated in 30% of CRCs overall, and in up to 90% of MSI-H tumors, where a notorious polyadenine repeat causes indel mutations, abolishing serine/threonine activity and phosphorylation of SMAD2/3. TGFβRI is less frequently mutated in CRC (5%–10%) and often co-occurs with the TGFβRII mutation. The germline variant TGFBR1*6A has been associated with increased CRC susceptibility. SMAD2 mutations appear in 6-8% of CRCs, also determining pathway disruption, while SMAD3 mutations are infrequent.^134^ In addition, the overexpression of negative regulators of the pathway, such as SMAD7, is observed in CRC and correlates with poor prognosis.

#### Therapeutic strategies

Given the prevalence of the TGF-*β*-SMAD4 dysregulation in CRC and its key role in promoting tumorigenesis, targeting this pathway may constitute an attractive therapeutic option. Dysregulation of the TGF-*β*-SMAD4 axis most frequently occurs as SMAD4 deletion or loss-of-function mutation and is, therefore, precluded from direct pharmacological targeting. However, efforts have focused on blocking the pro-tumorigenic arm of TGF-*β* that results from SMAD4 loss, and on taking advantage of SMAD4 loss through synthetic lethality. TGF-βRI-targeting small-molecule inhibitors have made it furthest into the clinic. Vactosertib has been evaluated in a single-arm Phase I/II clinical trial in combination with ICI blockade (NCT03724851) for pretreated MSS mCRC, where TGF-*β*-driven immune evasion has been documented,^34^ and has shown anti-tumor response and a 15.8-month median overall survival, with manageable toxicity.^33^ Studies that include CRC patients are ongoing with other TGF-βRI inhibitors. These include the SMIs galunisertib (NCT05700656, NCT02688712), LY3200882 (NCT02937272), GFH018 (NCT05051241), and the fusion protein Bintrafusp Alfa (NCT02517398). GFH018 demonstrated a safe toxicity profile in a Phase I trial in patients with solid tumors.^36^ Likewise, galunisertib demonstrated a 32% complete response rate and well-tolerability when added to a neoadjuvant regimen in locally advanced rectal cancer patients in a single-arm Phase II trial. Bintrafusp alfa, a bispecific agent composed of a TGF-*β*-binding, TGF-βRII-based fragment and anti-PD-L1, was tested in a Phase I clinical trial of pretreated MSS CRC patients but showed a lack of efficacy.^35^ NIS793 is a monoclonal antibody that blocks TGF-*β* and has been evaluated in a Phase 2 trial for mCRC alone or in combination with ICI blockade on top of standard-of-care regimens (NCT04952753) as second-line therapy; however, the trial was halted owing to safety concerns around NIS793. An alternative group of therapies that have been explored in the context of SMAD4-deficient tumors takes advantage of synthetic lethality; however, these approaches are still preclinical. A genome-wide CRISPR screen identified the small GTPase RAB10 as a synthetic lethal gene in SMAD4-altered CRC, suggesting that RAB10 inhibition could represent a viable strategy.^139^ Similarly, BRD2/4 and AURKA loss have been described as synthetically lethal in SMAD4 (Shi et al., 2020).^140^ Overall, therapeutic strategies that address SMAD4-induced TGF-*β* dysregulation are still largely unexplored, yet offer a new therapeutic angle that could improve the course of treatment for many CRC patients.

## Epigenetics

### Background

Beyond aberrant signal transduction cascades, which result from gene- and protein-level dysfunction, alterations of the epigenome also contribute to CRC progression. Many epigenetic variations can be considered driver events, often upstream of genetic and signaling cascades, thus precipitating disease. Advances in detection and sequencing technologies have made it possible to turn various epigenetic attributes from observations of tumor biology into useful diagnostic and prognostic biomarkers. Further, their potential reversibility positions epigenetic variations as attractive therapeutic targets, with recent strides made in translating their potential to preclinical and clinical models.

#### DNA methylation

DNA modifications can underlie significant disease-causing changes in gene expression. DNA methylation of cytosine residues at CpG dinucleotides, regions of DNA containing cytosine followed by guanine, is a pervasive chemical modification performed by DNA methyltransferases that serves as a mechanism for transcriptional repression. Most CG-rich areas in the genome are methylated; however, “CpG islands” consist of areas with high CG content that are often unmethylated. CpG islands are found in approximately 70% of human gene promoters.^141^ In CRC, CpG islands commonly undergo hypermethylation in the promoters of critical tumor suppressor genes. These repressive modifications have been identified in various genes involved in key signaling pathways discussed previously, including Wnt/β-catenin pathway genes such as APC and AXIN2 and the Wnt repressors DKK2/3, SFRP1/2/5, WIF1, and SOX17; the DNA repair genes MLH1 and MGMT, as well as MSH2 in Lynch syndrome-associated tumors; and the PI3K/AKT/mTOR antagonist PTEN, resulting in their transcriptional silencing.^142-149^ Promoter hypermethylation has been proposed as a successful diagnostic marker and prognostic indicator. In one study, hypermethylation of APC, MGMT, RASSF2A, and WIF1 had a positive predictive value of 90.6%, highlighting the utility and rigor of multi-target profiling in accurately identifying disease.^144^

In contrast, DNA hypomethylation refers to the loss of methylation relative to healthy tissue. Genome-wide hypomethylation in CRC has been identified at sites encoding transposable DNA known as long interspersed nuclear elements (LINEs), which are associated with increased chromosomal instability. LINE hypomethylation in polyps was found to be a significant predictor of future tumor development, suggesting that methylation screening is a powerful risk stratification tool.^150^

A variety of drugs targeting DNA methylation have been investigated as potential therapeutic agents for disease. DNA methyltransferase inhibitors (DNMTi) aim to relieve hypermethylation and transcriptional repression of tumor suppressive genes. The best-characterized DNMTis are cytidine analogs, which incorporate into DNA in place of normal cytidine and prevent DNMT-induced methylation, as well as bind and sequester DNMTs.^41^ The use of DNMTi is complicated by the abundance of hypomethylated DNA in tumors, which may become exacerbated by DNMTi, and significant off-tumor cytotoxicity associated with the drugs. Nevertheless, the safety and efficacy of DNMTi have been evaluated in the context of CRC, both as monotherapies and in combination with other treatments, such as radiation, chemotherapy, and other epigenetic drugs. One study screened and identified dozens of FDA-approved drugs that improved DNMTi activity in a CRC cell line model and downregulated oncogenic targets.^40^ These findings provide a potential therapeutic strategy that involves combination therapies against CRC. Additionally, several clinical trials have evaluated DNMTi in patient populations. Guadecitabine was well-tolerated with the chemotherapeutic agent irinotecan in a phase I/II study (NCT01896856). 5-Aza-4’-thio-2’-deoxycytidine is currently being evaluated in patients with solid tumors, including CRC, to determine a safe dose (NCT03366116). An active clinical trial is assessing changes in methylation in patients with resectable colorectal tumors after treatment with epigallocatechin-3-gallate, a non-cytidine analog DNMTi (NCT02891538). These recent and ongoing studies will provide insight into drug interactions, safety and tolerability, and the efficacy of DNMTi in CRC, which will be critical in informing new therapeutic strategies.

#### Histone modifications

Like DNA methylation, histone modifications are prevalent in the genome. Covalent modifications, including methylation, acetylation, phosphorylation, and ubiquitination, are deposited on histone tails, constituting a “histone code” that dictates gene expression by providing context-dependent information for chromatin modifiers and the transcriptional machinery. These reactions are catalyzed by histone-modifying enzymes, such as histone acetyltransferases (HATs) and deacetylases (HDACs).^151^ Dysregulation of standard histone modification patterns is frequently observed in CRC. Early work in the field relied on mass spectrometry, immunostaining, and site-specific chromatin immunoprecipitation (ChIP). These assays led to the discovery of global loss of H4K16ac and H4K20me3 in many tumor subtypes, including CRC.^152^ More recently, improvements in sequencing capabilities have identified a myriad of other aberrant histone modifications, including elevated H3K4me3 and H3K9me2, and reduced H3K9me3, H3K56ac, and H4K16ac.^153-155^ Many of these modifications, as well as the enzymes responsible for catalyzing their addition or removal from histone tails, also offer robust prognostic value and even successfully predict metastasis.^153^ ^,^ ^154^ ^,^ ^156^ Genome-wide changes in histone marks contribute to broad changes in cell signaling events, allowing for the aberrant activation of tumor-initiating and metastasis-promoting pathways discussed in previous sections.^157^

Despite recent advances, histone modifications remain challenging to screen in a high-throughput manner. Consequently, therapies targeting histone-modifying enzymes are less well-developed compared with those targeting DNA methylation. In that context, HDAC inhibitors (HDACi) aim to combat the transcriptionally activating hyperacetylation of histones at the promoters and enhancers of proto-oncogenes. FDA-approved HDACi have shown promise in treating hematological malignancies. In pre-clinical studies focusing on CRC, HDACi, including resminostat, belinostat, and panobinostat, have shown potent anti-tumor efficacy.^41^ Clinical trials are also in progress to evaluate HDACi. One active Phase II study investigated the HDACi chidamide in combination with the CapeOX chemo-immunotherapy regimen (NCT06709885). Previously, vorinostat was found to be well-tolerated in combination with a 5-FU chemotherapy regimen in metastatic tumors; however, its efficacy was poor, perhaps highlighting the need for therapies focused on early-stage disease.^158^ Drugs targeting other histone-modifying enzymes, such as methyltransferases (HMTs) and demethylases (HDMs) are not currently under clinical investigation for CRC, but are being evaluated in blood cancers (NCT03603951, NCT05627245, and NCT06357182).

#### Noncoding RNAs

RNA presents another point of epigenetic regulation beyond the level of DNA, contributing to regulatory aberrations in tumors. While mRNA serves as an intermediate link between DNA and protein in the context of genetic information transfer, it represents a small minority of total RNA in cells. In contrast, noncoding RNAs (ncRNAs) constitute a class of molecules with a range of different functions, including those involved in protein synthesis (tRNAs and rRNAs), and those that play a regulatory role (miRNAs, siRNAs, lncRNAs, etc.). ncRNAs are involved in every stage of genetic regulation, including chromatin remodeling, transcription, post-transcriptional processing, translation, and post-translational control.^159^

MicroRNAs (miRNAs), an important class of regulatory elements in cancer, are short, single-stranded RNA molecules that repress target mRNAs through complementary binding and sequestration or degradation.^159^ ^,^ ^160^ Many miRNAs are differentially expressed in CRC, including miR-31, miR-183, miR-17-5p, miR-18a, miR-20a, miR-92, miR-143, and miR-145, with some miRNA expression correlating with prognosis.^160^ miRNAs have been implicated in the dysregulation of Wnt, PI3K, and KRAS signaling.^42^ ^,^ ^161-164^ Inhibition of miRNAs restored proper Wnt signaling in cell lines and preclinical mouse models.^161^ ^,^ ^163^ In CRC cell lines, radiosensitivity was increased upon miRNA therapy, associated with reduced PI3K activity, highlighting a potential avenue for improved treatments in the future.^161^ Several clinical trials are currently evaluating miRNAs as diagnostic biomarkers of early disease and tools for patient stratification (NCT06351384, NCT06654622, NCT05346757, NCT02466113, NCT06342440, NCT06738225).

Clinically, miRNA therapy is in its early stages. One notable trial (NCT01829971) used a miR-34a mimic in patients with advanced tumors, including CRC. While the drug initially demonstrated tolerability and some anti-tumor activity, the trial was ultimately terminated owing to serious adverse events and immune-mediated toxicity in five patients, resulting in four deaths.^164^ Challenges associated with miRNA therapy include poor delivery to the cancer site and a lack of target specificity. In contrast, small interfering RNAs (siRNAs) are synthetic double-stranded analogs that offer improved specificity, with six therapies receiving FDA approval to date.^42^ An active trial is investigating tumor-infiltrating lymphocyte (TIL) therapy with siRNA-mediated suppression of PD-1 (NCT05902520). Similarly, APN401 is an siRNA targeting the immunosuppressive Cbl-b in peripheral blood mononuclear cells, with a recently completed trial in patients with advanced disease (NCT03087591). NBF-006 is an experimental siRNA targeting GSTP, a modulator of MAPK and PI3K signaling, which exhibited favorable anti-tumor efficacy in a pre-clinical lung model and recently completed a phase I clinical trial in lung, pancreatic, and CRC patients (NCT03819387).^43^ In addition to miRNAs and siRNAs, long non-coding RNAs (lncRNAs) are another major regulatory component in CRC. lncRNAs have been implicated in the regulation of Wnt, p53, TGF-*β*, AKT, and MAPK signaling cascades.^165^ The lncRNAs HOTAIR and MALAT1 are associated with poor prognosis in patient populations.^166^ ^,^ ^167^ While lncRNA-based therapies are not yet under clinical investigation, several recent and ongoing trials have sought to use lncRNAs for diagnosis and prognostic prediction (NCT06427278, NCT06531902, NCT06307249, NCT06534242, NCT04729855, and NCT06432413).

## Discussion

A central obstacle in advancing CRC therapeutics is the heterogeneity of the disease, which is evident both across patient populations and within individual tumors. Highlighted in this review, there is significant diversity in the molecular and genetic profile of each CRC, which continues to undermine one-size-fits-all treatment strategies. Importantly, the signaling pathways implicated in CRC do not function alone; rather, they exist within a network characterized by extensive pathway crosstalk and feedback regulation, which contributes significantly to tumor adaptability, disease progression, and therapeutic resistance. Several key examples underscore the complexity of these interactions. The Wnt/β-catenin and MAPK/ERK pathways demonstrate substantial crosstalk in CRC. Increased Wnt signaling represses the MAPK pathway through transcriptional regulation of proto-oncogenes such as MYC, while MAPK signaling may further potentiate *β*-catenin activity through phosphorylation events that promote nuclear localization and transcriptional function.^168^ A recent analysis of CRC patient data revealed that the metastatic gene signature associated with high MAPK and low Wnt signaling correlated with poor survival.^169^ Likewise, PI3K/AKT signaling is frequently activated downstream of oncogenic RAS, linking MAPK pathway dysregulation to enhanced cell survival and proliferation.^170^ Similarly, interactions between the Wnt and GUCY2C signaling axes further illustrate the layered regulatory architecture of CRC biology. Activation of Wnt signaling suppresses the expression of guanylin and uroguanylin, the endogenous ligands of GUCY2C.^74^ Loss of GUCY2C signaling subsequently promotes genomic instability, dysregulated proliferation, and metabolic reprogramming, thus accelerating tumor progression. This convergence of pathways ultimately contributes to aggressive tumor behavior and may partially explain resistance to therapies directed at individual signaling foci.

Bridging the gap between experimental discovery and clinical implementation represents an additional and persistent challenge. Widely used preclinical CRC models, while foundational to mechanistic and translational research, incompletely capture the cellular, molecular, and microenvironmental intricacies of human disease. Compounding these challenges is the pervasive issue of resistance to targeted therapies. Increasing resolution of CRC signaling networks has revealed not only actionable vulnerabilities but also the remarkable plasticity of malignant cells, which can evade therapeutic pressure through pathway redundancy, adaptive rewiring, or acquired genetic alterations. These observations underscore the need for adaptive and combinatorial treatment paradigms that anticipate tumor evolution rather than respond to it retrospectively.

This review outlines the complex network of signaling pathways that drive the development and progression of CRC. This review summarizes the interplay of biological pathways, genetic alterations, and epigenetic mechanisms in CRC, with particular emphasis on both current and emerging therapeutic targets. There are many emerging therapeutics being investigated, from miRNAs to vaccines to immune modulators, which offer promising avenues for improving outcomes in this highly lethal disease. The findings summarized here highlight the imperative for further research to prioritize the discovery of effective targets and agents that can circumvent therapeutic resistance and provide a roadmap for ongoing and future progress in CRC management. Indeed, this review underscores the need for a more nuanced, precision-driven strategy to develop effective individualized treatments for this morbid disease process.

## Data Availability

Data sharing is not applicable to this article, as no new data were created or analyzed in this study.
